# Effect of Beverages on the Hardness and Tensile Bond Strength of Temporary Acrylic Soft Liners to Acrylic Resin Denture Base

**Published:** 2013-12

**Authors:** A Safari, M Vojdani, S Mogharrabi, N Iraji Nasrabadi, R Derafshi

**Affiliations:** a Dept. of Prosthodontics, School of Dentistry, Shiraz University of Medical Sciences, Shiraz, Iran; b Dental Biomaterial Research Center, Dept. of Prosthodontics, School of Dentistry, Shiraz University of Medical Sciences, Shiraz, Iran; c Dept. of Prosthodontics, School of Dentistry, Jondi Shapour University of Medical Sciences, Ahvaz, Iran; d Dentist, Member of Student Research Committee, School of Dentistry, Shiraz University of Medical Sciences, Shiraz, Iran

**Keywords:** Beverage, Hardness, Tensile Bond Strength, Soft Liner

## Abstract

**Statement of Problem:** Two potential problems commonly identified with a denture base incorporating a resilient liner are failure of the bond between acrylic resin and soft liner material, and loss of resiliency of the soft liner over time. Since patients may drink different beverages, it is important to evaluate their effects on physical properties of soft lining materials.

**Purpose:** The objective of this in vitro study was to evaluate the effect of different beverages on the hardness of two temporary acrylic-based soft lining materials and their bond strength to the denture base resin.

**Materials and Method: **For the hardness test; a total of 80 rectangular specimens (40mm×10mm×3mm) were fabricated from a heat-polymerized polymethylmethacrylate. Two commercially auto-polymerized acrylic resin-based resilient liners; Coe-Soft and Visco-gel were prepared according to the manufacturers’ instructions and applied on the specimens. For the tensile test, 160 cylindrical specimens (30mm×10mm) were prepared. The liners were added between specimens with a thickness of 3 mm. The specimens of both soft liners were divided into 4 groups (n=10) and immersed in distilled water as the control group, Coca-Cola, 8% and 50% ethanol. All groups were stored in separate containers at 37^o^C for 12 days. All beverages were changed daily. The hardness was determined using a Shore A durometer and tensile bond strength was determined in a ZwickRoell testing machine at a cross-head speed of 5mm/min. The results were analyzed using two-way ANOVA.

**Results: **There was no significant interaction between the soft liners and the drinks for both hardness (*p*= 0.748) and bond strength (*p*= 0.902). There were statistically significant differences between all drinks for both hardness (*p*< 0.001) and bond strength (*p*< 0.05).

**Conclusion:** Within the limitations of this study, it seems that drinking Coca-Cola and alcoholic beverages would not be potentially causing any problems for the temporary acrylic soft liners.

## Introduction

Resilient soft lining materials can be helpful for the ma-nagement of patients with removable prosthesis who are unable to bear the hard denture base due to thin underlying mucosa, resorption of the residual ridge, severe undercuts and heavy and unequal distribution of occlusal loads [1-5]. Short-term-use soft liners can be employed as tissue conditioners, functional impression materials and temporary reliners of ill-fitting removable dentures [6-7]. These materials can also be used as the interim liners in the healing periods after implant placement [8]. 

**Table 1:** List of materials used in this study

**Product**	**Type of polymerization**	**Powder/** **Liquid Ratio**	**Manufacturer**
Meilodent	Heat-polymerized denture base polymer	23.4g : 10ml	Heraeus Kulzer, Hanau, Germany
Coe-Soft	Auto-polymerized acrylic resin-based resilient liner	11g : 8ml	GC America Inc., Alsip, Ill
Visco-gel	Auto-polymerized acrylic resin-based resilient liner	3g : 2.2ml	Dentsply, De Trey GmbH, Konstanz, Germany

Soft liners can be either heat-polymerized or auto-polymerized [3, 9] and they are usually provided as powder and liquid. The powder consists of polyethylmethacrylate (PEMA) and the liquid contains ethyl alcohol (as solvent) and an aromatic ester (di-butyl phthalate) as the plasticizer agent which is responsible for maintaining material softness [8, 10]. 

The efficiency of these materials is based on their cushioning effect and they lose their resiliency and become harder after clinical use. The temporary nature of these types of materials is because, in their clinical use, the alcohol and the plasticizer leach out from their structure and water or saliva is absorbed by them which eventually lead to the loss of viscoelasticity and their compliance [11-13]. 

Softness is a desirable property of resilient liners. Their optimum thickness has been reported to be approximately 2.5 to 3 mm to provide good shock absorption [14-15]. Another serious problem with these materials is bond the failure between the soft liner and the denture base. Any other desirable properties of a denture liner would not be beneficial, unless a good bond to the denture base is achieved. Other problems with soft liners include contamination and accumulation of microorganisms, plaque and calculus formation, poor tear and tensile strength [3, 8, 14-16]. 

Several studies have been carried out about the effects of water and denture cleansers on the properties of soft lining materials [7, 9, 17-18]. But there are few published articles in regard to the effect of beverages on the hardness and bond strength of resilient soft liner materials. Therefore, the aim of this study was to evaluate the effect of different beverages on the hardness and tensile bond strength of 2 acrylic auto-polymerizing temporary soft liners.

The null hypothesis is that the hardness values of the soft liners stored in beverages are the same as those of specimens immersed in distilled water and increasing the hardness does not change the bond strength.

## Materials and Method

In this in vitro experimental study, two commercially available acrylic resin-based temporary soft liners were chosen for evaluation. The brand of the resilient liners and the denture base resin material, their manufacturers, powder/liquid ratio and type of polymerization are listed in Table 1.

All materials, used in this study, were prepared according to the manufacturers’ instructions. A total of 80 rectangular specimens with a cross-sectional area of 40mm ×10 mm and thickness of 3mm were fabricated from heat-polymerized PMMA as the denture base material for the hardness test. These specimens were prepared by placing two metal plates (stainless steel) upon each other in a conventional denture flask with the aforementioned dimensions. The metal plates were invested in hard but flexible silicone rubber (LasticXtra; Kettenbach, Eschenburg, Germany) to facilitate the removal and the replacement of the plates and the specimens while maintaining the shape.

**Figure 1a:**
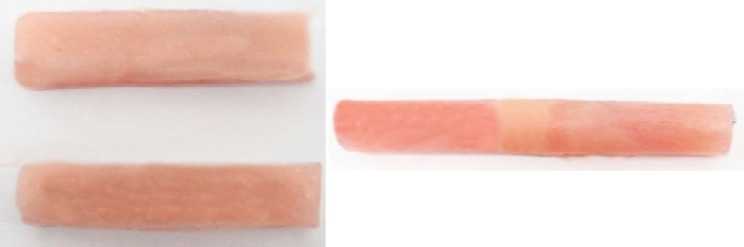
Specimens ready for hardness test **b** Specimens ready for tensile test

The upper metal plate was removed, denture base resin material was mixed and packed into the mold while the other metal plate was still present in the mold to act as a spacer and maintain 3mm space for the resilient lining material. The flask was placed under pressure in a standard flask press (No.01001; Teledyne Hanau. Buffalo; NY, USA) for 15 minutes; and denture base material was cured in a water bath at 75oC for 9 hours. After polymerization, processed denture base resin plate was removed from the flask and was trimmed. The metal spacer was then removed from the mold, PMMA block was placed back into the mold and temporary soft liner was packed against PMMA into the 3-mm space available on the block and the flask was placed under pressure in the flask press for 15 minutes. The specimens were removed from the flask and any flash was trimmed with a sharp blade No: 15 (Swann Morton; England) (Figure 1).

The specimens of both soft liners were divided into 4 groups (n=10) and were immersed in distilled water 

as the control group, Coca-Cola (Neysun shargh Co; Mashhad, Iran), 8% and 50% ethanol. All groups were stored in separate containers at 37^o^C, for 12 days. All beverages were exchanged daily. It is reported that 3.2 doses are consumed daily by a regular drinker and each dose lasts for 15 minutes. The 24-hour- storage time simulates one month of regular drinking [19]. Thus, the 12-day-immersion period in this study represents one year consumption of that beverage. The hardness was determined using a Shore A durometer tester (The Shore Instrument & Mfg Co Inc.; Freeport, NY, USA), which was calibrated according to ASTM D2240 and the results were reported in Shore units.

For the tensile test, 10 pairs of cylindrical specimens (30mm in height and 10mm in diameter) were prepared for each group by investing two stainless steel dies in front of each other and a 3-mm-thick spacer between them. The PMMA cylinders were fabricated in the flasks as previously described for the hardness specimens. Afterwards, the spacers were removed from the mold and the soft liner materials were mixed, packed in-to the flasks and were placed under pressure for 15 minutes. The specimens were then removed from the flask and any flash was trimmed with a sharp blade No: 1.

Tensile bond strength was determined using Zwick/Roell testing machine (ZwickRoell; Germany) when the failure have occurred and the magnitude of the force was recorded. The bond strength was calculated as maximum load (N) divided by the cross-sectional area (mm^2^) and reported in mega Pascal (MPa).

Two-way ANOVA and Tukey HSD post hoc tests were used to analyze the data. All tests were performed at a preset alpha level of .05 (α=.05) using statistical software (SPSS version 11.0 SPSS Inc.; Chicago, Ill).

## Results

There was no significant interaction between soft liners and drinks for both hardness (*p*= 0.748) and bond strength (*p*= 0.902) as indicated by the 2-way ANOVA (Tables 2 and 3).

**Table 2:** Two-way ANOVA results for comparison of hardness values

**Source**	**SS**	**df**	**MS**	**F**	**P**
Soft liner	0.086	1	0.086	0.010	0.922
Drink	4103.871	3	1367.957	153.346	<.001
Soft liner x drink	10.891	3	3.63	0.407	0.748
Error	642.294	72	8.921		
Total	39773.46	80			

**Table 3:** Two-way ANOVA results for comparison of hardness values

**Source**	**SS**	**df**	**MS**	**F**	**P**
Soft liner	0.001	1	0.001	3.188	0.078
Drink	0.238	3	0.079	424.947	<.001
Soft liner x drink	<.001	3	3.55	0.191	0.902
Error	0.013	72	<.001		
Total	14.761	80			

The mean and standard deviation values of hardness and bond strength of resilient liner materials, after immersion in the drinks, are shown in Table 4.

Comparison of the hardness and bond strength values between the two materials, in each drink separately and in total; did not show any statistically significant difference.

There were significant differences in the hardness of the specimens immersed in different drinks (Table 4). A significant increasing trend was observed in the hardness values of both soft liners. The results of the hardness test revealed that the mean hardness values (SD) of both Coe-Soft and Visco-gel was the least in 50% ethanol, followed by the specimens immersed in 8% ethanol, then Coca-Cola, and was the highest in distilled water (Table 4). Furthermore, comparing hardness of the specimens stored in each drink with the control group, there were significant differences between water and 50% ethanol (*p*< .001), water and 8% ethanol (*p*< .001), and also between water and Coca-Cola (*p*< .001). 

There were also significant differences in the bond strength values of the specimens stored in different drinks (Table 4). A significant decreasing trend was seen in the bond strength values of both materials. The results of the bond strength test demonstrated that the mean bond strength values (SD) of both soft liners was maximum in 50% ethanol, followed by the specimens immersed in 8% ethanol, then Coca-Cola, and minimum in distilled water (Table 4). Moreover, comparison of the bond strength values of the specimens stored in each drink with the control group as well as hardness results, showed significant differences between water and 50% ethanol (*p*< .001), water and 8% ethanol (*p*< .001), and also between water and Coca-Cola (*p*< .001).

**Table 4:** Mean (standard deviation) of the hardness (Shore Units) and Tensile bond strength (MPa) of the soft liners after 12 days immersion in different drinks

	**Hardness**				**Bond Strength**			
	
	**50% Ethanol**	**8% Ethanol**	**Coca-Cola**	**Water**	**50% Ethanol**	**8% Ethanol**	**Coca-Cola**	**Water**
Coe-Soft	12.43^Aa^ (2.84)	16.52^Ab^(1.51)	23.28^Ac^(3.87)	31.31^Ad^(3.37)	0.500^Aa^(0.011)	0.452^Ab^(0.011)	0.410^Ac^(0.014)	0.351^Ad^(0.012)
Visco-gel	13.08^Aa^(2.42)	15.46^Ab^(1.55)	24.08^Ac^(4.23)	31.18^Ad^(2.87)	0.494^Aa^(0.013)	0.443^Ab^(0.012)	0.408^Ac^(0.014)	0.345^Ad^(0.017)
Total	12.76^Aa^(2.59)	15.99^Ab^(1.58)	23.68^Ac^(3.97)	31.25^Ad^(3.05)	0.497^Aa^(0.012)	0.447^Ab^(0.012)	0.409^Ac^(0.014)	0.348^Ad^(0.015)

**Different superscripted uppercase letters indicate statistically different means within each column.**

Different superscripted lowercase letters indicate statistically different means within each row.

## Discussion

This in vitro study investigated the effect of different beverages on the hardness and tensile bond strength of two acrylic auto-polymerizing temporary soft liners. The results of this study rejected the null hypotheses so that the hardness values of the soft liners stored in beverages would be lower than those of specimens immersed in distilled water and with an increase in the hardness, bond strength would decrease.

Theoretically, liners should distribute functional stresses on the residual ridges evenly and should also absorb energy during mastication to reduce the transmitted loads to the mucosa [20]. During clinical use, the hardness of the soft lining materials changes and subsequently makes them ineffective [21]. In some investigations immersion in different solutions increased the hardness of the soft liners [18, 22-23]. It can be assumed that the plasticizer leaches out and the liquid is absorbed and these procedures would be responsible for the increase in the hardness [22]. However, depending on the formulation of the material and the duration of immersion, an increase or decrease in the hardness may be observed [21]. In our study, storage in Coca-Cola, 50% ethanol and 8% ethanol decreased the hardness of the resilient lining materials in comparison to the control group.

In this study, 50% ethanol and 8% ethanol decreased the hardness more than the Coca-Cola. This may be related to the presence of ethanol. It is known that ethanol acts as a plasticizer. In one study, it was shown that large amount of ethanol may accelerate degradation of a photo-activated soft lining material [24]. This deteriorating effect of ethanol on hardness is in agreement with other studies [21, 24]. This might indicate that the patients’ alcohol consumption could cause damage to soft lining materials [21]. 

The hardness values in our study were not in agreement with some other investigations [17, 25-26]. This difference may be due to the thickness of the specimens, periods of immersion and different solutions tested [27-29]. 

The water absorbed by a denture liner material has both direct and indirect damaging effect on its bonding to acrylic resin. The water absorbed may indirectly decrease the bond strength by causing plasticizer to leach out. The reduced plasticizer content will increase the stiffness and will reduce the cushioning effect of the liner material [30]. This would result in the vulnerability of the bond since external loads are transmitted directly to the bond site rather than being absorbed by the liner [17, 29]. The water may also percolate directly to the bond site leading to the swelling and consequently to the stress formation at the interface [29]. 

Craig [31] suggested that storage in water did not affect the bonding of denture liners to PMMA. By roughening the PMMA surface before bonding; it would approximately double the adhesion values of resilient liners.

Yanikogtlu and Denizoglu [9] reported that tensile bond strength of Visco-gel was increased with time in water. Since the powder of Visco-gel is a PEMA and no bonding agents are needed to achieve a bond with acrylic resin; they suggested that this could have occurred because of the leaching out of the plasticizer. This, in turn, resulted in increased hardness, thus resulting in mechanical bonding and chemical adhesion between soft liner material and acrylic resin [32]. 

The results of the present study did not agree with those of Craig [31] and Yanikogtlu and Denizoglu [9]. These dissimilarities might be due to the different parameters used in each study such as acrylic resin, storage time and solutions and cross-head speed of the testing machine.

The prominence of the present study was to investigate the influence of laboratory immersion in different beverages and solutions at 37^o^C on the hardness and bond strength of two temporary soft lining materials to simulate the mouth conditions and the clinically relevant regimens. As the hardness and bond strength values in water were the highest and the lowest respectively, it seems that consumption of Coca-Cola and the alcoholic beverages by the patients is not substantial and they would not cause significant deleterious effects on the hardness and bonding properties of temporary soft lining materials compared to water.

In clinical condition, however, these materials are subjected to additional changes in the hardness that might be related to the temperature fluctuation and the pH changes [21]. Some studies have shown that the deterioration of the soft lining materials was faster in clinical use than in immersion studies which had used artificial saliva and distilled water [33-34]. 

Further in vivo clinical investigations on the soft lining materials are required to determine the level of bond strength, hardness (softness) and other properties which are indispensable for effectiveness of these materials.
